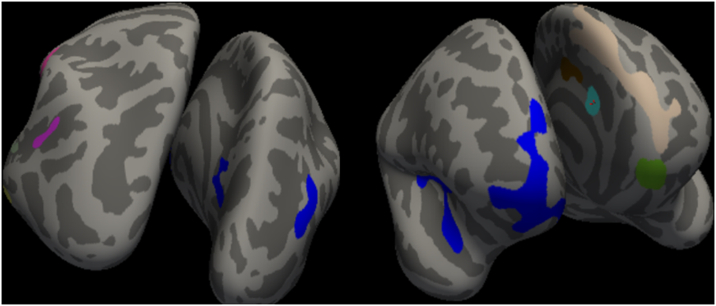# Increased Activity of the Glymphatic System in First-Episode Psychosis: Relationship With Cortical Thinning

**DOI:** 10.1016/j.jaacop.2026.04.004

**Published:** 2026-04-23

**Authors:** Emily Alder, Kahiau Among, Colton A. Olexa, Kristen R. Hoskinson, Whitney I. Mattson, Eric E. Nelson, Musa Yilanli

**Affiliations:** a Nationwide Children’s Hospital, Columbus, Ohio; b The Ohio State University College of Medicine, Columbus, Ohio

**Keywords:** glymphatic, ALPS, first-episode psychosis, neuroimaging, synaptic pruning

## Abstract

**Objective:**

First-episode psychosis (FEP) in pediatric onset is a pivotal window for preventing chronic disability and optimizing long-term outcomes. Improved understanding of psychosis biomarkers is essential to clarify the pathophysiology, guide individualized intervention, and improve prognostication for patients. We evaluated the glymphatic system (GS) as a potential mechanistic biomarker and assessed whether developmental processes influence GS function and cortical thinning in a cohort of FEP adolescents. We also explored the impact of age on GS function and cortical thinning.

**Method:**

A total of 21 FEP patient research participants (PRP) were recruited from a Psychiatry and Behavioral Health unit. The experimental battery included clinical and neuropsychological assessments and a magnetic resonance imaging session in which both T1 and diffusion-weighted sequences were acquired. PRP were compared to a normative set of 42 community control participants (CCP) from the same scanner. Across both groups, analysis along the perivascular space (ALPS), an approximation of glymphatic activity, was generated from the diffusion images, and whole-brain cortical thickness was compared between groups using Freesurfer software and statistical models.

**Results:**

Consistent with previous findings, significant and widespread reductions in cortical thickness were evident in the FEP population relative to the CCP. However, in contrast to expectations, ALPS was significantly elevated in PRP, suggesting increased GS functioning. Cortical thinning partially explained ALPS.

**Conclusion:**

In contrast to the few published studies in adults with schizophrenia, which have found significant reductions in ALPS, these results suggest that pediatric PRP with FEP have increased activation of the GS. Our findings suggest that this may relate to excessive cortical thinning in some brain regions (a hypothesis that we are currently exploring), although the directionality of this relationship is unclear. These findings may further indicate to a differential GS response in early vs late phases of disease or other differential pathologies related to development or disease phase, and to a differential role of GS and cortical thinning across groups.

## Plain language summary

This study examined the difference in brain waste clearance for adolescents with first-episode psychosis compared to adolescents with no history of psychosis symptoms. The study discusses synaptic pruning as a potential explanation for differences in waste clearance activity.

Early detection and treatment of first-episode psychosis (FEP) can have a substantial impact on quality of life, social functioning, and long-term prognosis. FEP is a crucial stage in the onset of severe psychiatric disorders, including psychotic spectrum disorders.^1^^,^^2^ For this population, developing reliable biomarkers is essential, as they serve to elucidate the underlying pathological mechanisms, guide treatment decisions, and facilitate more individualized care.^3^^,^^4^

In recent years, several studies have suggested that dysfunction in the glymphatic system (GS) may contribute to symptoms in chronic psychiatric conditions, such as schizophrenia. The GS, first described just over a decade ago, is responsible for clearing waste from the brain by facilitating cerebrospinal fluid (CSF) flow through periarterial spaces, mixing with interstitial fluid, and removing proteins, toxins, and metabolic waste through perivenous pathways.^5^, ^6^, ^7^ Dysfunction in this system has been linked to a variety of neurologic and neuropsychiatric disorders.^8^, ^9^, ^10^, ^11^ Recently, a magnetic resonance imaging (MRI)–based index of GS activity was proposed that incorporates diffusion tensor image analysis along the perivascular space (DTI-ALPS).^12^ Although it is not a comprehensive measure, several studies have used the ALPS as a proxy for glymphatic function.

Most previous studies in chronic neurological and psychiatric populations have reported reductions in the ALPS, which may indicate hypofunctionality of the GS and, consequently, a buildup of toxins in the brain. Adults diagnosed with long-term psychosis have been found to have lower DTI-ALPS indices compared to control groups,^13^, ^14^, ^15^ and similar findings have been reported in an early psychosis sample.^16^^,^^17^ Additionally, for people with schizophrenia, APQ-4, a component of GS, was linked to neuroinflammation, indicating glymphatic activity as a possible biomarker.^18^^,^^19^ However, all of these studies were in adults, which may not capture a critical time point for the evolution of psychosis, given that psychotic disorders often emerge earlier than adulthood. Findings of dysregulation of the GS even in early phases of psychosis are important, as these consistent findings suggest that hypofunction of the GS may contribute to psychiatric symptoms.

Assessments of GS function based on disease duration and medication exposure demonstrate how GS function interacts with different treatment experiences for people with psychosis. Regarding disease duration, some evidence of a positive association between the upregulation and enlargement of the blood–cerebrospinal fluid barrier and chronic psychosis compared to early onset suggests targeted regulation of brain homeostasis and response to peripheral events along disease duration.^20^ Additionally, medication exposure may affect glymphatic function in psychosis, as one of the few studies to report lower ALPS in psychosis was performed in people exposed to antipsychotics.^15^ Furthermore, an important unresolved issue is whether glymphatic functionality is impaired before or after symptoms. Although hypoactivation of the ALPS has been reported in many psychiatric disorders, the specific neurological mechanism, directionality, and timing in relation to symptoms and treatment have not been well characterized. By extending these studies to a younger age at an earlier phase of illness, we hope to shed additional light on these issues.

Most clinical ALPS studies have been conducted in adults with chronic diseases. This is important to address because of the potential relationship to, and differences from, adults that are related to age, disease duration, and medication exposure. For example, although it is suggested that glymphatic dysfunction precedes psychosis symptoms, the study population was restricted to adults, and it did not measure the potential differences in glymphatic function due to differences in developmental stages.^15^ Some studies have found that developmental changes in glymphatic function occur through adolescence. For example, ALPS index has been shown to peak in the fourth decade of life, followed by a decline with advanced aging^21^; however, a steep increase in glymphatic function was captured between the ages of 10 and 20 years, indicating an adolescent period of a comparatively rapid increase in glymphatic activity. These changes could reflect the physiological maturation of the glymphatic system. Alternatively, developmental changes could also occur because of different waste clearance demands generated by synaptic pruning in childhood and adolescence. This could be a critical mediating factor in pediatric psychosis, as excessive pruning during adolescence is well documented in FEP^22^^,^^23^ and may contribute to the demands on GS function.

In addition, GS activity follows a circadian rhythm, with high activity levels during sleep and relative quiescence during active daytime hours.^24^^,^^25^ In addition to increased demands related to pruning, sleep demands increase during adolescence, and sleep quality is often disrupted for adolescents with psychosis symptoms.^26^^,^^27^ Thus, 2 factors that drive GS function—pruning and sleep—are increased during the adolescent period, and both are disrupted in youth with psychosis.

These factors underscore the importance of considering GS in an adolescent sample. The goals of the present study are to evaluate glymphatic function using the ALPS method for patient research participants (PRP), a pediatric population undergoing the first episode of psychosis, relative to an age-matched sample of community control participants (CCP) with no history of psychotic symptoms. A secondary objective was to assess potential moderating factors on ALPS, including cortical thickness and sleep dysregulation as independent and potentially interactive factors with the group on ALPS, and illness duration in the FEP group.

## Method

### Study Design and Oversight

This was a single-site, observational cohort study of adolescents with FEP conducted at a hospital in a midwestern region of the United States. Recruitment occurred between May 2023 and May 2025. An institutional review board approved the protocol and all procedures. Given the minor status of participants (12-17 years of age), youth provided informed assent, and a parent or legal guardian provided informed consent before any study activities. The procedures adhered to the Declaration of Helsinki and local regulations for research with children.

### Participants

#### Screening and Enrollment

A total of 24 consecutive youth meeting clinical criteria for psychosis were screened and enrolled from a psychiatric inpatient unit (n = 22) and psychosis specialty clinic (n = 2). This represented each participant’s first episode of psychosis, and most were not medication-naive, having initiated antipsychotic treatment shortly before baseline study procedures. Caregivers were invited to participate in providing parent-report measures. Complete MRI data could not be obtained for 3 PRP, who were subsequently excluded from these analyses. PRP with missing MRI data either did not complete the DTI portion of the scan or declined participation in the scan task after enrollment. The total sample of individuals with FEP in the present study was 21.

#### Inclusion and Exclusion Criteria

Participants were eligible for the study by meeting the following criteria: (1) age 12 to 17 years; (2) clinical diagnosis of a psychotic disorder consistent with *DSM-5-TR* spectrum conditions at first episode; (3) ufficient capacity to assent and comply with study procedures; and (4) no contraindications for an MRI scan and able to complete an MRI scan without sedation. Participants were excluded for any of the following: (1) substance-induced psychosis or a psychotic disorder due to a general medical condition; (2) known intellectual disability (clinical history of IQ <70 or equivalent documentation), or global developmental conditions precluding valid assessment; (3) neurological or medical conditions likely to affect brain structure and function or to confound neuroimaging and assessment (eg, epilepsy/seizure disorder, moderate to severe traumatic brain injury or head trauma with loss of consciousness, brain tumor, CNS infection, stroke, significant sensory impairment such as deafness or uncorrected visual impairment); (4) contraindications to MRI (eg, non-removable ferromagnetic implants, braces, or devices incompatible with MRI, claustrophobia unresponsive to behavioral supports); and (5) primary psychosis related to recent drug intoxication. These exclusions mirror the criteria and early-intervention samples that separate affective (A-FEP) and non-affective (NA-FEP) psychoses.

#### Diagnostic Procedures and Clinical Characterization

A licensed child and adolescent psychiatrist or a supervised trainee conducted baseline diagnostic intake interviews and chart reviews. *DSM-5-TR* diagnoses were established using the clinician-administered Kiddie Schedule for Affective Disorders and Schizophrenia (K-SADS),^28^ supplemented with collateral history from caregivers and review of medical records. Cases were subsequently classified into affective first-episode psychoses (A-FEP; n = 8), including psychotic major depression and bipolar disorder with psychotic features, vs non-affective first-episode psychoses (NA-FEP; n = 13), encompassing schizophrenia spectrum and related disorders. When clinically indicated, short-term follow-up evaluations were obtained to stabilize diagnoses after the acute episode, typically within 1 to 2 months, consistent with comparable cohorts. Given the potential for diagnostic instability in early psychosis, particularly in youth, these groupings should be interpreted with caution.

### Control Sample

The present study included a community control (CC) sample of 72 adolescents from a study on the trajectory of neurodevelopment, supplying a larger existing dataset of scans. An institutional review board approved the study procedures and analyses. From the control sample, an age- and sex-matched subset of 42 individuals was selected for comparisons. The final sample comprised 63 participants (21 FEP and 42 CC). Sample descriptives for age at the time of the MRI scan, sex, duration of illness (DOI), symptom severity, duration of antipsychotic medication exposure (APE) in days, and the total dose taken over the total days on medication (mean dose, in milligrams) at the time of scan are displayed in Table 1. All tables and figures are original works and have not been adapted or republished from another source.

**Table 1:** Descriptive Statistics for First-Episode Psychosis and Community Control Groups

Variables	CC (n = 42)	FEP (n = 21)
Age at scan, y, mean (SD)	15.77 (2.63)	16.27 (1.11)
Sex, n^a^		
Male	26 (61.9^a^)	16 (76.2^a^)
Female	16 (38.1^a^)	5 (23.8^a^)
**FEP variables (n = 21)**	**Min–Max**	**Mean (SD)**
DOI	3–1,694	306.52 (485.91)
PANSS symptom severity	48–152	107.1 (25.96)
Antipsychotic exposure	2–302	36.24 (66.61)
Mean dose (mg)	0.66–325	73.15 (104.66)

Note: CC = community control group; DOI = duration of illness; FEP = first-episode psychosis; PANSS = Positive and Negative Syndrome Scale.

a Value reported as percentage.

### Study Procedure and Measures

The full battery included a series of neuropsychological tests and self-report questionnaires, a blood sample, and an MRI scan. The control sample was drawn from a larger pool of participants acquired on the same scanner with identical diffusion MRI sequence parameters (discussed below). Age- and sex-matched CCP were selected at a 2:1 ratio to the PRP. Matching was performed using the MatchIt package in R, using the optimal pair-matching method.

#### Sleep Quality

Sleep quality was measured with the Pediatric Sleep Questionnaire (PSQ), a 22-item parent-report screening tool designed to assess pediatric sleep-disordered breathing, snoring, daytime sleepiness, and behavioral correlates. To calculate the scores, the total positive responses were divided by the total reported responses, excluding unknown and no responses. Each “yes” is scored as 1 and “no” as 0, with “don’t know” responses treated as missing. Scores range from 0 to 1, with a commonly used clinical cutoff of >0.33 indicating increased risk of sleep-disordered breathing. Higher scores indicate more disordered sleep.

#### Duration of Illness

Duration of Illness (DOI) was defined as the number of calendar days from onset to the date of MRI acquisition (the first usable scan), regardless of treatment status. Onset was the first day that *DSM-5-TR* psychosis criteria were met and symptoms either persisted for ≥7 days or prompted emergent evaluation, hospitalization, or antipsychotic initiation. Threshold symptoms included hallucinations, delusions, disorganized thinking/speech that impaired communication, or grossly disorganized/catatonic behavior; episodes occurring exclusively during intoxication/withdrawal or due to a general medical condition were excluded, and prodromal/attenuated phenomena alone did not contribute. DOI was calculated using multiple sources, including youth and caregiver interviews anchored to the K-SADS, electronic health record documentation (first psychosis note, emergency department/inpatient note), and medication start dates, with consensus adjudication when sources diverged. When the precise onset date could not be established, the earliest reliably documented timepoint across sources was used. Durations were analyzed continuously (days).

#### Symptom Severity

Symptom severity was defined as the general psychopathology score on the Positive and Negative Syndrome Scale (PANSS), a clinician-administered report used to quantify symptom severity profiles.

### MRI Protocol

Structural (T1-weighted) and diffusion-weighted MRI (dMRI) scans were collected for all participants using a 3 Tesla Siemens Prisma scanner equipped with a 64-channel head coil during a single dedicated research MRI session. Structural scans used a magnetization prepared rapid gradient echo (MPRAGE) sequence (TR = 2,500 milliseconds, TE = 1.81 milliseconds, field of view [FOV] = 240 × 256 mm, voxel size = 0.80 mm^3^, flip angle = 8°). Diffusion-weighted images (TR = 4,200 milliseconds, TE = 89 milliseconds, FOV = 2,160 mm, voxel size = 1.71 mm^3^, flip angle = 90°) with 102 directions were acquired.

### DWI Data Processing

Diffusion weighted imaging underwent a standardized pipeline for TBSS preprocessing until tensor images were obtained. First, the c4d tools program was used to pad images for subsequent trimming and masking with FSL’s brain extraction tool (bet). FSL’s eddy_openmp was used to correct radial distortions due to motion. Advanced Normalization Tools (ANTs) were then used for nonlinear registration of b0 to T1 images and subsequent transformation of DWI images. After distortion correction, the diffusion and ALPS analyses followed a standardized process for tensor fitting, ROI association, and projection placements. FSL’s DTIFIT was used to fit tensors from corrected images, generating fractional anisotropy (FA) maps and directional tensor images (xx, yy, zz, xy, xz, yz). Then, coordinates based on an established region of interest (ROI) placement were transformed into FMRIB58 template space, and 5-mm ROI spheres were placed at those coordinates.^29^ DTIFIT transformation matrices were then applied to nonlinearly warp the participants’ tensor maps to the FMRIB58 FA template by applying the nonlinear warp using FSL’s vecreg function, which accounts for the effects of warping on the calculation of tensor direction. Finally, from the tensor image, mean values of the xx and yy directions in the projection ROI and xx and zz directions in the association ROI were calculated. The ALPS index was determined by dividing the average diffusivity in the xx-direction within the projection and association fibers by the average diffusivity in the yy-direction of the projection fibers and the zz-direction of the association fibers.^29^ The formula follows:

$$\text{DTI}\;\text{ALPS}=\frac{mean\;\left(Dxxproj,Dxxassoc\right)}{mean\;\left(Dyyproj,Dzzassoc\right)}$$

Two ALPS indices corresponding to the right and left brain hemispheres were generated.

### Structural Preprocessing and Cortical Thickness

The primary purpose of incorporating cortical thickness into our analyses was to assess whether cortical thinning associated with developmental pruning is related to GS activity. If so, group differences in ALPS may be expected because of the greater amount of thinning associated with psychosis during the adolescent period.^30^ The recon-all command from Freesurfer was used to generate 3-dimensional (3D) cortical parcellations and volumetric segmentation from all T1 images. Two models were used to evaluate cortical thickness. First, the average cortical thickness for the left and right hemispheres for all participants was calculated across all cortical regions, resulting in a single cortical thickness score for the left and right hemispheres. Second, to assess whether specific brain regions were associated with ALPS indices, voxelwise models were created, enabling group comparisons for specific cortical regions.

### Statistical Analysis

All primary statistical analyses were conducted using IBM SPSS Statistics Version 28. Prior to analyses, all continuous variables were assessed for normality using the Shapiro–Wilk test and for homogeneity of variance using the Levene test. One variable (PSQ) violated normality because of an outlier; a log transformation was applied for subsequent analyses. Because the transformed variable still failed to meet normality assumptions, both Pearson and Spearman correlations were used, depending on distribution, to examine bivariate relationships across the full sample and within each group.

An independent-samples *t* test was first used to assess overall group differences for ALPS indices, average cortical thickness (left and right hemispheres), and PSQ scores (Table 2). Then, using the averages across hemispheres for ALPS and cortical thickness, an analysis of covariance (ANCOVA) was run to model the impact of group, cortical thickness, PSQ, sex, and age on ALPS. To maintain statistical power, the sex covariate was excluded from the final ANCOVA model (Table 3); however, these results are presented in the Supplemental Material (Table S1, available online). Another ANCOVA was run with interaction terms (group × cortical thickness and group × age) to test the impact of group on ALPS as cortical thickness and age change (Table 3). Then, stepwise regression was run to isolate the influence of group and cortical thickness on ALPS. Bivariate correlations among ALPS indices, cortical thickness, sex, age, and PSQ scores were examined separately within the CC and FEP groups, and across the entire sample (Table S2, available online).

**Table 2:** Group Differences in Along the Perivascular Space, Cortical Thickness, and Sleep Quality

Variable	FEP mean (SD)	CC mean (SD)	*t* (*df*)	*p*
Left ALPS	1.598 (0.18)	1.504 (0.16)	–2.11 (61)	.039∗
Right ALPS	1.686 (0.16)	1.538 (0.17)	–3.33 (61)	<.001∗
LH cortical thickness	2.503 (0.06)	2.609 (0.08)	5.35 (61)	<.001∗
RH cortical thickness	2.483 (0.06)	2.577 (0.08)	4.68 (61)	<.001∗
PSQ score	.072 (0.04)	.051 (0.03)	–1.95 (26.35)	.062

Note: ALPS = Along the Perivascular Space; CC = community control group; LH = left hemisphere; PSQ = Pediatric Sleep Questionnaire; RH = right hemisphere.

∗Significant at .05, ∗∗significant at ≤.001 (2-tailed).

**Table 3:** Analysis of Covariance Results for Group, Cortical Thickness, Age, Sleep Quality, and Interaction Terms

Variable	*df*	*F*	*p*
Group	1, 50	.35	.558
Average cortical thickness	1, 50	2.94	.093
Age	1, 50	.49	.489
PSQ score	1, 50	.99	.325
*R*^*2*^ = 0.149; adjusted *R*^*2*^ = 0.080			
**ANCOVA model results with interaction terms**			
**Variable**	***df***	***F***	***p***
Group	1, 57	.02	.886
Average cortical thickness	1, 57	1.3	.259
Age	1, 57	.23	.634
Group × average CT	1, 57	.19	.662
Group × age	1, 57	1.08	.302
*R*^*2*^ = 0.164; adjusted *R*^*2*^ = 0.091			

Note: ANCOVA = analysis of covariance; CT = cortical thickness; LH = left hemisphere; PSQ = Pediatric Sleep Questionnaire; RH = right hemisphere.

To evaluate differences within the FEP group, correlations between ALPS indices and DOI, PANSS scores, APE, and mean dose were conducted (Table S2, available online). In addition, an ANCOVA was run to model the impact of cortical thickness, PSQ, DOI, PANSS, APE, mean dose, sex, and age on ALPS for the FEP group (Table S3, available online). To explore differences in ALPS indices, an ANCOVA was conducted to model DOI, PANSS, PSQ, age, and sex (Table S4, available online).

Freesurfer Group Descriptor (FSGD) files were used to compare the FEP and CC groups on several contrasts with the glymphatic ALPS average value as a covariate. The first contrast assessed whether there was a difference between the FEP and CC groups, regressing out the effect of the glymphatic ALPS value. The second contrast tested whether the effects of ALPS values on cortical thickness differed between groups. The final contrast tested whether glymphatic value had an effect regardless of group membership (effect of cortical thickness on glymphatic). The analyses evaluated significant differences in cortical thickness between the FEP and CC groups. Then differences in cortical thickness were evaluated while accounting for glymphatic function. In addition to the hemisphere averages, the differences in cortical thickness within brain regions were assessed.

## Results

### Group Differences in ALPS Index, Cortical Thickness, and PSQ for FEP and CC

**Figure 1:**
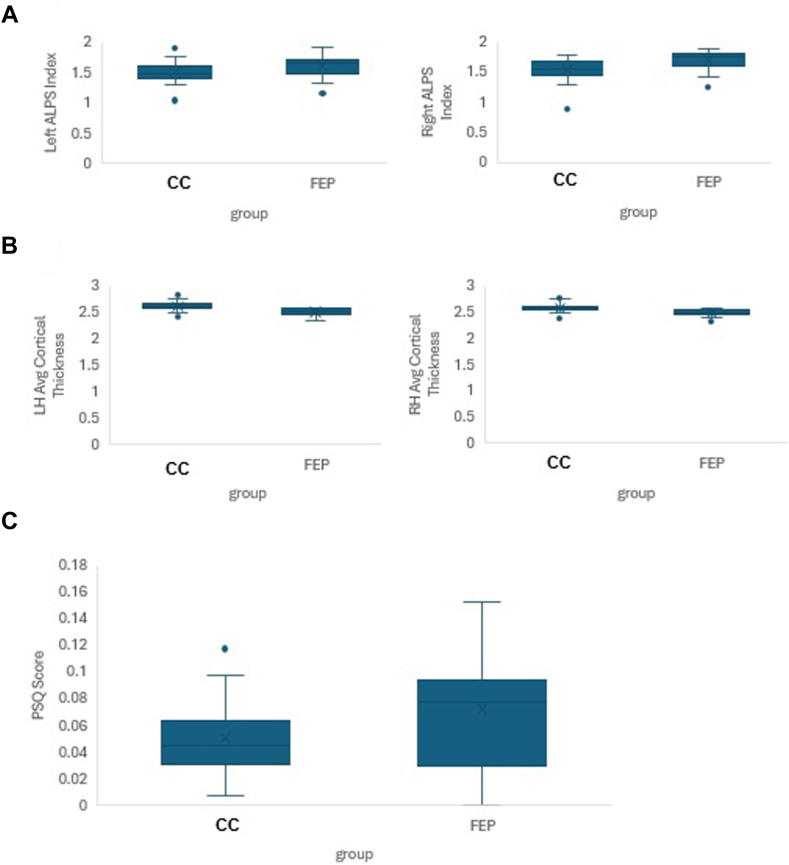
Overall Group Differences in Study Measures ***Note:****(a) FEP had significantly higher ALPS indices in the left and right hemispheres than did the CC. (b) FEP had significantly lower cortical thickness in the left and right hemispheres than did the CC. (c) FEP had non-significantly higher PSQ scores than did the CC. Error bars represent 95% CIs. Values outside the error bars represent outliers >2 SD. ALPS = along the perivascular space; CC = community control group; FEP = first-episode psychosis group; LH = left hemisphere; PSQ = Pediatric Sleep Questionnaire; RH = right hemisphere.*

**Supplementary Figure S1:**
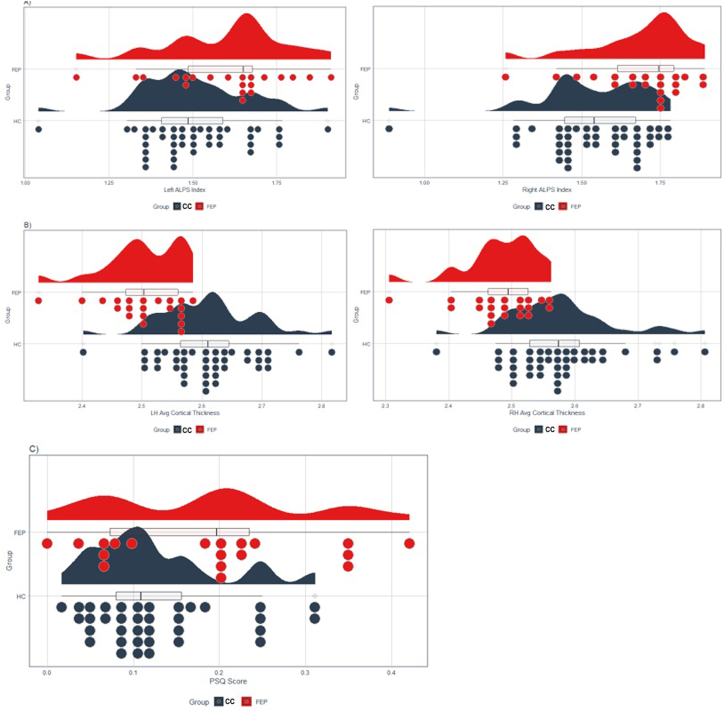


Our examination of group differences by variable is depicted in Figure 1. Individual data points for the group comparison are available (Figure S1, available online). There were significantly higher left and right ALPS values for the FEP group than for the CC group (left: *p* = .039; right: *p* = .001). Average cortical thickness across hemispheres was significantly lower in the FEP group than in the CC group (left: *p* < .001; right: *p* < .001), as summarized in Table 2. In addition, the FEP group had lower cortical thickness in the following left hemisphere regions: superior frontal gyrus (superiorfrontal) (*t* = –6.02, *k* = 2,888, *p* = .001, xyz = [–12.8, 62.6, 14.2]), inferior temporal gyrus (inferiortemporal) (*t* = –8.31, *k* = 2,240, *p* = .001, xyz = [–44.6, –14.3, –36]), postcentral gyrus (postcentral) (*t*= –5.98, *k* = 1,168, *p* = .001, xyz = [–44.2, –9.6, 14.1]), and the medial orbitofrontal cortex (medialorbitofrontal) (*t* = –4.29, *k* = 439, *p* = .001, xyz = [–5.6, 55.8, –20.2]). Additional regions were significant at *p* < .05 corrected,^31^ and are summarized in Table 3. Figure S2, available online, provides the Freesurfer brain image with highlighted areas of differential cortical thickness. Although PSQ scores were higher in the FEP group than in the CC group, they were not significantly different, given that scores were not normally distributed (*p* = .062) (Figure 1c, Table 2.).

### Relations Among Variables for FEP and CC groups

The first ANCOVA showed no significant differences for group, average cortical thickness, PSQ, sex, or age in predicting average ALPS (*ps* > .05). The second ANCOVA, adding interaction terms, showed no significant impact of group on average ALPS as cortical thickness and age changed (*p* > .05). Stepwise regression was used to isolate the group and cortical thickness effects on ALPS. Group was the only significant predictor of the average ALPS (*F*_1,61_ = 8.52, *p* = .005, *R*^*2*^ = 0.12). The addition of average cortical thickness did not significantly improve the model (*ΔR*^*2*^<.01, *p* > .05). ANCOVA results are displayed in Table 4. Freesurfer analyses showed that as ALPS increased, there was significantly lower cortical thickness in the right precentral gyrus (precentral) across groups (*t* = 28.13, *k* = 149645, *p* = .002, x,y,z = [37.8, 3.7, 14.5]) (Table 3).

**Table 4:** Freesurfer Differential Cortical Thickness

LH region	*t*	*k*	*p*	x, y, z
Superiorfrontal	–6.02	2,888	.001	–12.8, 62.6, 14.2
Inferiortemporal	–8.31	2,240	.001	–44.6, –14.3, –36
Postcentral	–5.98	1,168	.001	–44.2, –9.6, 14.1
Medialorbitofrontal	–4.29	439	.001	–5.6, 55.8, –20.2
Supramarginal	–5.08	728	.005	–56.6, –26.5, 28.5
Caudalanteriorcingulate	–4.41	567	.01	–8.7, 9.1, 35.2
Fusiform	–6.42	425	.014	–36.4, –18.2, –26.9
Bankssts	–4.52	479	.016	–48.3, –40.6, 1.2
Inferiorparietal	–3.57	331	.034	–41, –53, 12.6
Rostralanteriorcingulate	–4.6	230	.048	–9.5, 34.6, 16.8
**RH region**	***t***	***k***	***p***	**x, y, z**
Medialorbitofrontal	–7.72	3,170	.002	5, 37.9, –22.4
Insula	–5.89	1,946	.002	37.1, –7.6, –10.6
Inferiorparietal	–5.3	679	.002	39.4, –80.3, 27.4
Fusiform	–6.21	657	.004	29.4, –67.7, –8.6
Precuneus	–4.84	573	.016	14.4, –56, 20.7
Differential cortical thickness, including ALPS covariate				
RH precentral	28.13	149,645	.002	37.8, 3.7, 14.5

Note: ALPS = along the perivascular space; LH = left hemisphere; RH = right hemisphere.

The correlations between the left and right ALPS indices and cortical thickness, sex, age, and PSQ for the CC and FEP groups, both independently and across both groups, are depicted in Table S2, available online. There were significant correlations between right and left hemisphere cortical thickness and left ALPS in the total sample (*ps* < .05). This was followed up by examining whether age and sleep quality interacted with cortical thickness in predicting ALPS indices. The interaction between age and cortical thickness did not significantly predict either left or right ALPS (*ps* > .05). Furthermore, potential correlations between ALPS indices and DOI, PANSS scores, APE, and mean dose were explored for the FEP group. Mean dose negatively correlated with right and left ALPS (right ALPS *r* = –0.44, *p* = .049; left ALPS *r* = –0.633, *p* = .002), yet was non-significant in subsequent ANCOVA models examining mean dose on ALPS when the models included other variables (*p* > .05). Further details are described in Supplement 1, available online. DOI, PANSS, and APE were non-significant (DOI: left ALPS *r* = –0.21, *p* = .369; right ALPS *r* = – 0.03, *p* = .911; PANSS: left ALPS *r* = –0.190; *p* = .422, right ALPS *r* = 0.213, *p* = .367). These results are shown in Table S2, available online.

## Discussion

Previous studies have found hypofunctioning of ALPS in psychosis, suggesting that compromised GS and waste removal in the brain may contribute to psychotic symptoms. The present study used the DTI-ALPS method as a proxy for glymphatic function to assess the glymphatic function of adolescent PRP who experienced a first psychotic episode. Contrary to previous studies in adults, the ALPS indices were significantly higher for adolescent psychosis PRP compared to CC, indicating increased GS function in the psychosis group. To explore this discrepancy with the literature, we incorporated 3 other variables that may be uniquely important during adolescence and that are thought to contribute to the emergence of psychosis in adolescence: synaptic pruning, age, and sleep. However, we acknowledge that many other factors differ between pediatric and adult samples, including symptom acuity and duration of medication exposure. These and other factors may also contribute to differences in children and adults with psychotic disorders.

Prior studies have hypothesized that the consistent observation of greater loss of gray matter in adolescents with psychosis relative to community control participants may be the result of excessive pruning, particularly in the prefrontal cortex.^32^ This excessive pruning, particularly during adolescence, may in turn result in dysregulation of dopaminergic activity in the midbrain and basal ganglia. We speculated that higher ALPS during the adolescent period, which contrasts with findings of reduced ALPS index in adults, may also be a marker of this process in adolescents with psychosis. If this is the case, the DTI-ALPS index may be a viable radiologic marker of disease progression in youth. We tested this hypothesis by correlating measures of cortical thickness with the ALPS index at the individual and group levels. Compared to the CC group, the FEP group had lower average cortical thickness in the left and right hemispheres and reduced cortical thickness in several brain regions, including the medial orbitofrontal gyrus, inferior parietal gyrus, and fusiform gyrus. Although we cannot directly attribute this difference to pruning per se because of the cross-sectional nature of this study, this effect is consistent with greater pruning during the adolescent period in adolescents who experience psychosis.^33^

Given the significantly lower cortical thickness in the FEP group, we speculated that an increase in the ALPS index may reflect greater glymphatic activity to remove excess gray matter lost to excessive pruning in the FEP group. We found that average cortical thickness in both the left and right hemispheres was negatively associated with an ALPS index, as would be indicated if ALPS were to reflect pruning. However, the stepwise regression showed that cortical thickness did not significantly predict ALPS when group was included in the model, suggesting some independent variance associated with group. This may indicate that although cortical pruning may be contributing to the ALPS index in adolescence, different pressures on the glymphatic system function may also be present in youth with psychotic disorders. Although the ALPS remains a viable marker of cortical thinning, future studies with larger sample sizes, and preferably a longitudinal design, will be needed to assess this relationship more thoroughly. The Freesurfer analyses clarified the mixed findings between the correlation and stepwise regression models by showing that ALPS explained differences in cortical thickness, yet for a substantially narrower brain area than for group differences. Synaptic pruning partially explained the elevated GS activity in the FEP group, but additional factors influenced the relationship. This finding indicates that higher levels of cortical gray matter loss are partially associated with more activation of the GS. Moreover, the elevated glymphatic activity may demonstrate a physiological response to a period of structural turnover. This finding is not unexpected, given the role of GS as a system for removing protein and other waste products; however, the relationship needs further examination. In addition, age was examined as a factor of ALPS due to pruning in adolescence, but it was not a significant predictor.

In this study, we assessed the role of sleep problems on ALPS function. The GS is particularly active during sleep, and sleep disruptions may dysregulate it. Here, we found that the higher sleep problems in the FEP group were non-significant and did not predict ALPS. Sleep quality findings remain exploratory, as the PSQ proxy is limited. Nevertheless, in this dataset we did not find any relationship between sleep problems and ALPS activity that may explain the elevated ALPS index in the FEP group.

Finally, we examined the influence of DOI and total PANSS scores on ALPS, as previous studies found reduced waste clearance in the brain associated with more severe psychosis symptoms.^16^ The results demonstrated no significant differences in glymphatic function related to either the illness duration or PANSS scores. The novel age group in the study sample may explain the discrepancies with previous literature, as the youngest participant in other studies was older than the participants in the present study. The developmental period of the adolescent sample, as well as the early point of psychosis symptom progression, may be key differences in explaining the results.

The demonstration of heightened glymphatic activity in adolescents with first-episode psychosis is consistent with research connecting glymphatic function with biological processes in several psychiatric disorders, including anxiety, depression, sleep, and schizophrenia.^34^ Our findings add to this growing literature and suggest the potential of glymphatic function as a biomarker for early psychosis. These markers might enhance diagnostic accuracy and inform tailored intervention strategies.^35^ Interventions that enhance GS activity, such as optimizing sleep hygiene, promoting exercise, or ensuring adequate hydration, may constitute innovative adjunctive strategies in treating early psychosis, although these await empirical support.

In addition, exploring ALPS as a biomarker for excessive pruning can potentially affect the clinical implications of early psychosis treatment. If additional studies with larger longitudinal designs demonstrate a significant influence of ALPS on cortical thickness, then ALPS may be an important marker to track in adolescents who are experiencing psychosis or at risk for developing psychiatric disorders. Gray matter loss is a well-documented aspect of chronic psychiatric disorders such as schizophrenia, and has been linked to cognitive decline and positive and negative symptoms. As such, the ALPS may be a useful marker for therapeutic interventions (behavioral or pharmacological) that target gray matter atrophy. Conversely, it is also possible that increased activity in the GS may play a causal role in gray matter atrophy during adolescence and may be a target of future treatment approaches.

It is important to recognize limitations regarding the sample size, variable medication exposure, and the indirect ALPS measure. The relatively small sample size may have restricted the ability to identify more nuanced correlations among cortical thinning, sleep, and GS function. Although medication exposure was reduced, it was not eliminated, and the antipsychotic effects on GS are still inadequately defined.^15^ Moreover, the constrained sample size did not allow for stratifying effects between participants with antipsychotic medication exposure vs medication-naive participants. The lack of precise antipsychotic exposure at the time of the scan also limited the information about the participants at the time of data acquisition. Although there was little variation across subjects in the time of day when scans were acquired, the study did not control for circadian variability at the subject level. In addition, the ALPS index serves as an indirect proxy for glymphatic function,^12^ and subsequent research should use multimodal approaches, such as contrast-enhanced MRI or cerebrospinal fluid biomarkers, to more thoroughly assess GS activity. More extensive, longitudinal, and multimodal studies could elucidate whether GS alterations in youth function as protective mechanisms, early indicators of disease burden, or prospective therapeutic targets.

In summary, this study offers novel evidence about GS that, in contrast to adult patients, who consistently show decreased GS function, adolescents with FEP may show increased glymphatic activity. According to Hsiao *et al.* (2023) and Korann *et al.* (2024),^15^^,^^21^ this points to a developmental difference in which the adolescent brain may develop an early compensatory clearance response that decreases with the chronicity of illness. Clinically, these results highlight the importance of considering the developmental stage when interpreting GS biomarkers in psychosis. They also imply that interventions that aim to preserve GS integrity in the early stages of illness may have long-term neuroprotective advantages. Further research is warranted to examine whether supporting GS function during adolescence could alter disease trajectories and improve psychiatric outcomes.

## CRediT authorship contribution statement

**Emily Alder:** Writing – review & editing, Writing – original draft, Visualization, Resources, Project administration, Methodology, Formal analysis, Data curation, Conceptualization. **Kahiau Among:** Writing – review & editing, Visualization, Methodology, Formal analysis, Data curation. **Colton A. Olexa:** Resources, Data curation. **Kristen R. Hoskinson:** Writing – review & editing, Supervision, Resources, Methodology, Investigation. **Whitney I. Mattson:** Writing – review & editing, Visualization, Software, Resources, Methodology, Formal analysis. **Eric E. Nelson:** Writing – review & editing, Supervision, Resources, Investigation, Conceptualization. **Musa Yilanli:** Writing – review & editing, Supervision, Resources, Project administration, Methodology, Investigation, Funding acquisition.

## Figures and Tables

**Supplementary Figure S2:**