# Supplementary material for: Increased Activity of the Glymphatic System in First-Episode Psychosis: Relationship With Cortical Thinning
Source: JAACAP Open. 2026 Apr 23;4(4):691–700. doi: 10.1016/j.jaacop.2026.04.004 (PMC13420542; doi:10.1016/j.jaacop.2026.04.004)
Supplement: Supplementary Tables S1-S4 [file mmc1.docx]

**S1.**  Antipsychotic exposure and mean dose were examined as potential confounding variables and were tested for significant associations with Average ALPS for the First-Episode Psychosis group. Due to the small sample size (n=21), a base model was run, followed by models that included an additional variable. The base model included Average Cortical Thickness, Duration of Illness, Symptom Severity, Sex, and Age (as shown in Table S3). The first subsequent model included the base with antipsychotic exposure. The second subsequent model included the base with mean dose. Before analysis, the antipsychotic exposure and mean dose variables were transformed to address skewness. Antipsychotic Exposure was non-significant (*F*(1,12)=1.022, *p*=.332). Mean Dose was also non-significant (*F*(1,12)=3.057, *p*=.106).

| **a.** ANCOVA for Group, Cortical Thickness, Age, and Sleep Quality on Average ALPS | | | | |
| --- | --- | --- | --- | --- |
| Variable |  | df | F | *p*-value |
| Group |  | 1, 49 | .238 | .628 |
| Average Cortical Thickness |  | 1, 49 | 2.67 | .109 |
| Age |  | 1, 49 | .012 | .913 |
| Sex |  | 1, 49 | 1.22 | .274 |
| PSQ |  | 1, 49 | .884 | .352 |
| R² = .169; Adjusted R² = .084 | | | | |
| **b.** ANCOVA Results with Interaction Terms | | | | |
| Variable |  | df | F | p |
| Group |  | 1, 56 | .003 | .96 |
| Average Cortical Thickness |  | 1, 56 | 1.22 | .274 |
| Age |  | 1, 56 | .63 | .43 |
| Sex |  | 1, 56 | 1.59 | .212 |
| Group x Avg CT |  | 1, 56 | .11 | .741 |
| Group x Age |  | 1, 56 | 1.03 | .315 |
| R² = .187; Adjusted R² = .1 | | | | |

**Table S1.** ANCOVA results for Group, Cortical Thickness, Age, Sleep Quality, and Interaction Terms

**Note:** ALPS = along the perivascular space; ANCOVA = analysis of covariance; CT = cortical thickness; PSQ = sleep quality.

| a) Total | Left ALPS |  | Right ALPS |  |
| --- | --- | --- | --- | --- |
|  | r | *p*-Value | r | *p*-value |
| LH Cortical Thickness | -.29* | .02 | -.24 | .056 |
| RH Cortical Thickness | -.25* | .048 | -.23 | .085 |
| Age | .08 | .515 | -.08 | .511 |
| Sex | -.13 | .3 | -.256* | .043 |
| PSQ | .103 | .45 | .23 | .097 |
|  | | | | |
| b) FEP | Left ALPS index |  | Right ALPS index |  |
|  | r | *p*-Value | r | *p*-value |
| LH Cortical Thickness | -.27 | .233 | -.16 | .5 |
| RH Cortical Thickness | -.26 | .252 | -.22 | .341 |
| Age | .337 | .135 | .07 | .759 |
| Sex | -.209 | 362 | -.156 | .5 |
| PSQ | -.04 | .886 | .12 | .635 |
| PANSS | -.190 | .422 | .213 | .367 |
| DOI | -.21 | .369 | -.03 | .911 |
| Antipsychotic Exposure | -.07 | .764 | -.189 | .412 |
| Mean Dose | -.633** | .002 | -.44* | .049 |
|  | | | | |
| c) CC | Left ALPS index |  | Right ALPS index |  |
|  | r | *p*-Value | r | *p*-value |
| LH Cortical Thickness | -.14 | .368 | .02 | .893 |
| RH Cortical Thickness | -.09 | .555 | .04 | .783 |
| Age | -.003 | .985 | -.18 | .242 |
| Sex | -.046 | .774 | -.245 | .118 |
| PSQ | .13 | .444 | .16 | .361 |
| ** sig at the .01 level (2-tailed)  * sig at the .05 level (2-tailed) | | | | |

**Table S2.** Correlation Table for Cortical Thickness, Age, Sleep Quality, Symptom Severity, Medication, and ALPS Indices

**Note:** a) correlations for entire sample; b) correlations for the First-Episode Psychosis group; c) correlations for the community control group.

**Note:** ALPS = along the perivascular space; CC = community controls; LH = left hemisphere; PANSS = symptom severity; PSQ = sleep quality; RH = right hemisphere.

| **a.** ANCOVA for Duration of Illness and Symptom Severity on Left ALPS for First-Episode Psychosis Group | | | | |  |
| --- | --- | --- | --- | --- | --- |
| Variable |  | df | F | *p*-value | ηp² |
| DOI |  | 1, 12 | .553 | .471 | .044 |
| PANSS |  | 1, 12 | .431 | .524 | .035 |
| Age |  | 1, 12 | 1.16 | .304 | .088 |
| Sex |  | 1, 12 | 3.25 | .097 | .213 |
| PSQ |  | 1, 12 | - | .984 | - |
| R² = .307, Adjusted R² = .018 | | | | |  |
| **b.** ANCOVA for Duration of Illness and Symptom Severity on Right ALPS for First-Episode Psychosis Group | | | | |  |
| Variable |  | df | F | *p*-value | ηp² |
| DOI |  | 1, 12 | .001 | .973 | - |
| PANSS |  | 1, 12 | .411 | .533 | .033 |
| Age |  | 1, 12 | .001 | .976 | - |
| Sex |  | 1, 12 | 3.39 | .091 | .001 |
| PSQ |  | 1, 12 | .016 | .901 | .001 |
| R² = .243; Adjusted R² = -.073 | | | | |  |

**Table S3.** ANCOVA for Duration of Illness and Symptom Severity on Right and Left ALPS for First-Episode Psychosis Group

**Note:** **A)** ANCOVA results for First-Episode Psychosis group regarding Duration of Illness and Symptom Severity on Left ALPS Index. **B)** ANCOVA results for First-Episode Psychosis group regarding Duration of Illness and Symptom Severity on Right ALPS Index.

**Note:** ALPS = along the perivascular space; ANCOVA = analysis of covariance; DOI = duration of illness; PANSS = symptom severity; PSQ = sleep quality.

| Variable |  | df | F | *p*-value | ηp² |
| --- | --- | --- | --- | --- | --- |
| Average Cortical Thickness |  | 1, 11 | .06 | .818 | .005 |
| DOI |  | 1, 11 | .12 | .736 | .011 |
| PANSS |  | 1, 11 | .00 | .992 | .000 |
| Sex |  | 1, 11 | 2.36 | .152 | .177 |
| Age |  | 1, 11 | .20 | .666 | .018 |
| PSQ |  | 1, 11 | .012 | .915 | .000 |
| R² = .249; Adjusted R² = -.161 | | | | |  |

**Table S4.** ANCOVA results for FEP group regarding Cortical Thickness, Duration of Illness, Symptom Severity, Sex, Age, and Sleep Quality on Average ALPS

**Note:** DOI = duration of illness; PANSS = symptom severity; PSQ = sleep quality.
